# A Wireless 32-Channel Implantable Bidirectional Brain Machine Interface

**DOI:** 10.3390/s16101582

**Published:** 2016-09-24

**Authors:** Yi Su, Sudhamayee Routhu, Kee S. Moon, Sung Q. Lee, WooSub Youm, Yusuf Ozturk

**Affiliations:** 1 School of Electronic Information, Wuhan University, Wuhan 430072, China; 2 Department of Electrical and Computer Engineering, San Diego State University, San Diego, CA 92182, USA; sudhamayeeedu@gmail.com; 3 Department of Mechanical Engineering, San Diego State University, San Diego, CA 92182, USA; kmoon@mail.sdsu.edu; 4 Electronics and Telecommunications Research Institute (ETRI), Daejeon 34129, Korea; hermann@etri.re.kr (S.Q.L.); wsyoum@etri.re.kr (W.Y.)

**Keywords:** implantable biomedical sensor, brain-machine interfaces, wireless sensor networks, local field potential, stimulation

## Abstract

All neural information systems (NIS) rely on sensing neural activity to supply commands and control signals for computers, machines and a variety of prosthetic devices. Invasive systems achieve a high signal-to-noise ratio (SNR) by eliminating the volume conduction problems caused by tissue and bone. An implantable brain machine interface (BMI) using intracortical electrodes provides excellent detection of a broad range of frequency oscillatory activities through the placement of a sensor in direct contact with cortex. This paper introduces a compact-sized implantable wireless 32-channel bidirectional brain machine interface (BBMI) to be used with freely-moving primates. The system is designed to monitor brain sensorimotor rhythms and present current stimuli with a configurable duration, frequency and amplitude in real time to the brain based on the brain activity report. The battery is charged via a novel ultrasonic wireless power delivery module developed for efficient delivery of power into a deeply-implanted system. The system was successfully tested through bench tests and in vivo tests on a behaving primate to record the local field potential (LFP) oscillation and stimulate the target area at the same time.

## 1. Introduction

Neuroplasticity is an intrinsic property of the human central nervous system (CNS) and represents the ability of actively adapting to environmental pressures, physiological changes and experiences [1]. Neuroplasticity occurs either during normal brain development when people begin to process new sensory information or as an adaptive mechanism to reform neurological paths due to brain or spinal cord injury (SCI). Damage to the CNS affects at least two million people per year. Compensation for brain or spinal function loss occurs after CNS injuries, such as stroke [2] or SCI [3]. The result of this compensation may take place not only in the cortex, but also involves other subcortical parts [4]. Thus, systems that can interpret different levels of brain activity and use it to interact with prosthetic devices and computer systems have immense potential for applications in various fields.

Brain machine interfaces (BMI) provide a non-conventional communication link between the brain and physical devices by offering an alternative path effectively, bypassing the original pathway when it is no longer available. By decoding different patterns of brain activity into commands in real time, BMI can be used to control assistive devices, monitor the affective state of a patient during and after a rehabilitation session and help the brain to regain motor functionality. Although early BMI research concentrated on noninvasive methods that provide over the scalp measurements, the state of the art applications in prosthetic device control received the most benefit from electrodes implanted under the scalp [5]. In a pioneering study, two 96-channel intracortical microelectrode arrays were implanted in the motor cortex of a 52-year-old individual with tetraplegia [6]. After 13 weeks of training, with the goal of controlling an anthropomorphic prosthetic limb, the patient was able to perform robust seven-dimensional movements of the prosthetic limb routinely. Since there exists no cure for upper limb paralysis, resulting in complete damage to neuron-motor pathways after spinal cord injury or stroke, the successful experiments in controlling external prosthetic devices or robotic arms are a major step towards enabling the recovery of the patients suffering from complete loss of their extremities.

Patients carrying a traditional wired BMI system suffered from the bulky system size, which resulted in less mobility and high risks of infection that lead to inconvenient user experiences. Thus, the main innovation of the research we proposed is to present a high performance implantable BMI system that enables wireless data transmission and maintains low power consumption with novel features integrated into a compact-sized device design. Related works in developing wireless BMI systems emerging in recent years promote neuroplasticity development in addition to providing system-level solutions that enable brain signal recording with varies wireless data and power transmission techniques. In [7], the authors presented a Wireless Human ECoG-based Real-time BMI System (W-HERBS) that could provide 128-channel neural signal recording at a sampling rate of 1 k samples per second (SPS). The system used a set of two Bluetooth devices for wireless data transfer. W-HERBS was charged wirelessly using a wireless charging coil. In [8], a 64-channel electrocorticography (ECoG) record only system with a dynamic gain and sampling rate was presented. This study employed inductive coupling for wireless power transfer. The power consumption of the system was reported as 350 mW. The ECoG data were transferred wirelessly to a host PC.

Both systems reported in [6,8] only provide one-way (mono-directional) communication from the brain to an external processor, which stores, filters and classifies the recorded signals to make inferences. A bidirectional brain machine interface (BBMI) can both record and stimulate neural tissue, providing an unprecedented opportunity to reanimate paralyzed limbs through neuroplasticity. Neurochip-2 was among the first systems to offer a multichannel bidirectional brain machine interface [9]. Neurochip-2 provided three channels of real-time recording with configurable gain and front-end filters. In addition, Neurochip-2 also provided three output channels for inducing intracortical stimulation based on an autonomous decision-making algorithm running on board. Neurochip-2 enabled the recording of a wide variety of neural signals and studied the effect of electrical stimuli applied to a free-moving animal model. With sampling rates between 256 SPS and 24 kSPS, the power consumption of Neurochip-2 was reported between 284 mW and 420 mW based on the configuration of the system. A stimulation circuit could deliver ± 15 V and 10–200 μA current pulses. It is important to note that Neurochip-2 is a wearable system with offline recording and stimulation. In [10], a 32-channel closed-loop system that integrated optical stimulation and neural signal recording was presented. The system was able to record at a sampling rate of 12.5 kSPS per channel, perform online spike detection and deliver spike-triggered optical stimuli. The integrated online spike sorting algorithms could process 32 channels of data in 8 ms. A wireless Brain-Machine-Brain Interface system named PennBMBI [11] was a general purpose closed-loop BBMI with four recording channels at a sampling rate of 21 kSPS. PennBMBI also provided two channels of stimulation with a stimulation compliance voltage of ±12 V. In addition to neural signal recording and stimulation, inertial, temperature and force measurements can be reported via a body sensor network. In [12], a 16-channel (eight channels for recording and eight channels for stimulation) wireless portable system with both recording and stimulation capabilities was presented. More recently, a wireless headstage with 32-channel neural signal recording and up to 32-channel optical stimulation has been presented in [13]. The system was tested through in vivo experiments and reported average SNR of 17.0 dB with an FPGA-based spike detection and data compression algorithm. A detailed comparison between the wireless BMI systems mentioned above to the system presented in this work is provided in the Results section.

While we gain some success in controlling external prosthetic devices, restoring function to the patient’s actual limbs by inducing cortical plasticity to promote sensory motor recovery following stroke or incomplete SCI remains a challenge. Recent research suggests that users can learn to modulate their sensorimotor rhythm to issue control commands to physical devices [14]. Neural signals recorded using invasive, or to a limited degree, non-invasive, sense electronics can be processed for intent detection, and the outcome can be used for controlling external devices. Current research in implantable BMI partitions the system into internal and external components. While the electrode arrays and sense electronics are implanted, almost all computational functions are performed by external components. Further, according to [12], one must find a balance among the limited resources due to the small form factor. The studies mentioned in the above literature either lack the ability to integrate all functional components into a reasonably small-sized enclosure or have a satisfactory size, but not fully-bidirectional communication. The trade-offs between bandwidth, the number of recording and stimulation channels, power consumption, size and processing features have to be carefully considered when designing the hardware and software of an implantable BMI solution.

In order to conquer the challenges listed above, this study introduces a new implantable wireless BBMI that can be used for inducing cortical and spinal plasticity to promote the creation of new neural pathways. The system developed in this study is a multichannel, small form factor, low power and low noise solution for neuro-rehabilitation and assessing brain response to stimulation. The system can provide 32-channel of brain activity recording from implanted electrodes and four channels of stimulation to the brain or spinal cord based on a protocol submitted wirelessly to the device. The system utilizes a low power communication system on chip (SoC) that implements the micro Enhanced ShockBurst (*μ*ESB) protocol. Based on the brain activity report, the system can induce stimulation to the brain and record neural activity as a response to the stimulus. The system utilizes the ultrasonic power transfer technique developed in a collaborative study [15] for wirelessly charging an on board battery. The hardware and component-based software platform we developed were validated through in vivo tests and adapted by other research groups.

## 2. Bidirectional BMI Design Challenges

**Figure 1:**
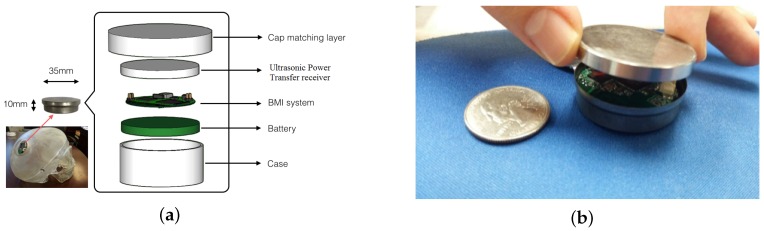
(**a**) Implantable bidirectional brain machine interface (BBMI) module in titanium casing with 3D representation. (**b**) Implantable BBMI module size in comparison with a quarter.

Due to the invasive nature of the BBMI systems, design spaces have to be confined to the following key issues during the design process:

Size: A compact design is needed so that the contact with vital brain tissues and the invasive system is kept to a minimum. The BBMI module developed in this study is encapsulated into a 35 mm in diameter, 10 mm in thickness titanium casing, as shown in Figure 1a,b. Inside the case, the ultrasonic power transfer receiver is placed on top of the BMI module while the 3.7-V Li-ion rechargeable battery is placed underneath. The headers shown on the PCB board are for debugging purposes only. Once the device is enclosed and sealed in the case, those debug headers will be removed.

Placement: Invasive BMI sense electrodes are inserted into the brain (intracortical electrode arrays) or sit on the surface of the brain just beneath the skull (cortical surface electrodes). The challenge in both cases is the placement of the electronics in close proximity to a vital organ. Both biocompatibility and risk of contamination are major research issues addressed by others in the field. Several placement methods have been proposed to address this challenge. In [16,17], only electrode arrays were implanted underneath the skull, while all other electronics were placed outside the body with a wired connection between the two units. In [18], the authors applied preclude , DuraGen , a silicon elastomer and methyl methacrylate around the inserted electrode to protect brain tissues from the risk of contamination. Data acquisition electronics and the battery module were enclosed in an aluminum housing, which was attached to the skull. In [19], the electrode, as well as the data acquisition electronics were implanted through a small burr hole in the skull and secured to the skull. In [20,21,22,23,24,25,26], only electrode arrays were implanted under the skull, while sealed recording electronics rested on the skull, but underneath the scalp. Placing the electronics between the skull and the scalp protected the brain from a direct thermal load, current leakage and contamination. In [27], the system with sensor arrays was placed inside a 50-mm craniotomy with the sensor side in direct contact with dura mater. Meanwhile, the antenna is placed between the skull and the scalp. The system presented in this study is intended to be a skull implant where part of the skull will be replaced with a titanium implant housing the electronics.

Communication interface: Invasive BMI systems require high standards for wireless interface due to the strict power and thermal constraints. Traditional methods such as Bluetooth do not satisfy the bandwidth and power limitations. Hence, proprietary protocols offering a high data rate with minimum power consumption are needed.

Power consumption: Power consumption is one of the crucial factors when designing an invasive BMI system. With lower power consumption, longer battery life could be achieved. Although wireless power transfer is used for charging the batteries, frequent charging is not desirable due to heat generation and the possibility of tissue damage due to electromagnetic or ultrasonic waves used for power transfer.

Thermal dissipation: Many components of implanted electronics will contribute to heat generation, which may affect the tissue. In [28], the heat flux of the implanted electronics is studied and measured to be 2.62 mW/cm${}^{2}$, which led to an acceptable temperature increase of less than one degree. The system developed in this study is enclosed in a titanium casing, which can be used as insulation due to its low thermal conductivity. Considering the fact that the system is designed to be deployed as part of the skull, power and thermal requirements are less strict compared with the systems that come in contact with brain matter.

In addition to the considerations discussed above, two different development paths have been chosen by researchers when designing implantable BMI solutions. One path aims to develop custom integrated circuit (IC) solutions that are optimized for size and power consumption. The other path utilizes commercial off-the-shelf (COTS) electronics to develop and offer a BMI solution. While custom IC solutions [29,30] might outperform some COTS solutions in terms of power and size, our design goal is to use the existing technology to advance the understanding of brain at a reduced cost and development cycle.

## 3. The Bidirectional BMI System Design

**Figure 2:**
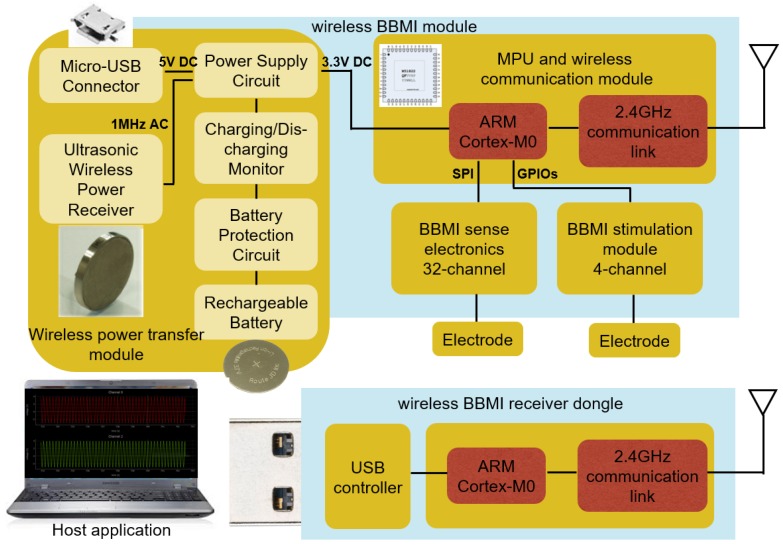
BBMI system block diagram including the implantable BBMI module, receiver dongle and host.

**Figure 3:**
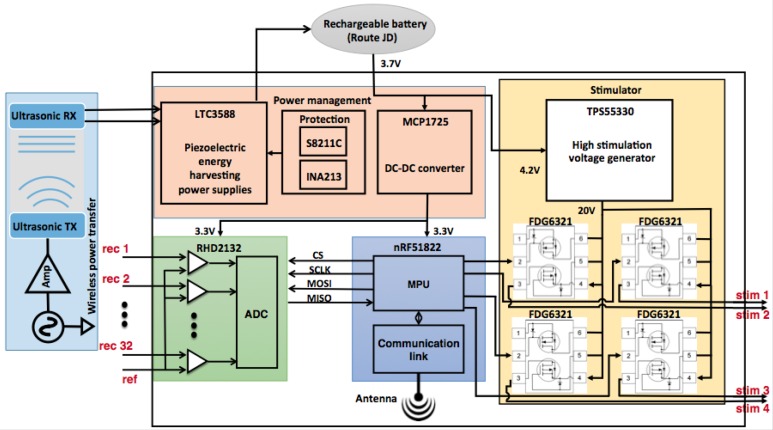
Implantable BBMI module circuit diagram and signal routing among the components.

In this study, a 32-channel, small form factor, low power and low noise BBMI system, providing excellent detection of a broad range of frequency oscillatory activities is designed and confined to the design spaces described in Section 2. The detailed BBMI system block diagram is given in Figure 2. The figure shows the implantable wireless BBMI module, the wireless BBMI receiver dongle and the host application. The core component of the BBMI system is a communication SoC with an embedded microprocessor unit (MPU), ARM-cortex M0. The processor is employed for controlling (1) wireless data transmission, (2) monitoring the charging circuit, (3) data acquisition and, finally, (4) stimulation configuration. Sense electronic module provides 32 input channels with a gain of 45.67 dB and small input referred noise. The stimulation module provides four output channels supporting unipolar 20-V stimulus with programmable frequency and pulse duration. The power module consists of the ultrasonic power receiver and the components for charge/discharge protection. Traditional USB charging is also available when ultrasonic power transfer is disabled. This component-based system is developed with modularity and extendibility in mind; in other words, providing standardized interfaces to third party hardware and software. The PCB board is 30 mm in diameter; while enclosed in the casing, this makes the whole system size 35 mm in diameter and 10 mm in thickness. Figure 3 shows the implantable wireless BBMI module circuit diagram with signal routing and information for each component.

### 3.1. Electrode

Electrodes are used for measuring the induced voltage when neurons are activated. For implantable BBMI devices, a microelectrode array (MEA) has been established as a bidirectional interface to sense extracellular neural activities and to provide electrical stimuli. Among different types of MEAs, Utah Array [31] is widely used for its excellent chronic stability. In this study, in vivo tests were performed on a primate whose motor cortex has been implanted with a typical high-impedance Utah Array electrode (Blackrock Microsystems, Salt Lake City, UT, USA). Although recording and stimulation are performed through the same MEA, the individual electrodes assigned for recording and stimulation are different due to circuit limitations. Work is in progress to offer both stimulation and recording through the same electrode via de-multiplexing recording and stimulation circuits to the same electrode.

### 3.2. Sense Electronics Module

Brain signals have been acquired using a digital electrophysiology interface chip from Intan Technologies (Intan Technologies, Los Angeles, CA, USA). This sense electronic module, as shown in Figure 3, has a fully-integrated electrophysiology amplifier array with an on-chip 16-bit analog-to-digital converter (ADC) and industry-standard serial peripheral interface (SPI). The analog front end has a fixed gain of 45.67 dB (192) and a programmable range for amplifier bandwidth selection. The amplifier low cut-off frequency is in the range of 0.02 Hz–1 kHz, and the high cut-off frequency is from 10 Hz–20 kHz. The low and high cut-off frequencies are in a broad range that is capable of capturing different brain signals. This enables a wide spectrum of recording from single unit recording local field potential (LFP) to high frequency spike detection. The chipset is a complete low-power electrophysiological signal acquisition system specifically designed for a dedicated brain signal recording system.

### 3.3. MPU and Wireless Communication Module

A low energy communication SoC by Nordic Semiconductor (nRF51822, Nordic Semiconductor, Oslo, Norway) provides the wireless interface between the sense electronics module and any host that can integrate 2.4-GHz protocol stacks. The SoC also integrates an ARM-cortex M0 processor as a central controlling unit to manage the system functions. The detailed circuit diagram and signal routing are shown in Figure 3. The nRF51822 communication SoC supports three data rates, 2 Mbps, 1 Mbps and 250 kbps. Depending on the target signal characteristics, the transmission of raw brain data requires a high throughput. In the case of LFP, a bandwidth of approximately 700 kbps is required for the transmission of 32 channels of BMI signals sampled at 1 k samples per second (SPS) with 16-bit resolution. Enhanced ShockBurst (ESB), a wireless protocol provided by Nordic Semiconductor, is able to send packets every 1.2 ms with 32 bytes of data inside each packet. ESB is sufficient for recording and transmitting LFPs with a moderate sampling rate and number of channels. Besides the proprietary protocols provided by Nordic Semiconductor, an enhanced *μ*ESB protocol can be used to communicate brain signals to a host computer when large data throughput is desired. The proprietary protocol *μ*ESB can transmit data in a connectionless manner, which is ideal for spike detection applications. With *μ*ESB, the wireless BBMI system developed in this study is able to transmit a signal with up to 3 kSPS for 32 channels. If the sampling rate is increased, the number of channels recorded will be reduced. On the receiver side, a dongle with an nRF51822 chip and a USB controller (nRF51 dongle, Nordic Semiconductor, Oslo, Norway) are programmed to receive data over the wireless link. The overall component-based architecture provides a system-level flexibility to integrate different sensors into one sensor network. In [32], we presented a body area network node with an integrated EMG sensor and a nine-axis inertial sensor. The body area network node could readily be integrated with the electrophysiological interface presented in this study; this will extended the sensorimotor feedback capabilities of the system. The body area network could host seven such network nodes deployed on the same person.

### 3.4. Stimulation Module

Applying stimulation to the brain or spinal cord is used for the treatment of certain conditions that affect CNS. The system presented in this paper, shown in Figure 3, features four-channel differential or unipolar high voltage stimulation channels for cortical stimulation or spinal cord stimulation. In the differential stimulation configuration, two electrodes are paired in one stimulation channel generating two 20-V high voltage monophasic pulses, which could form a biphasic current pulse depending on different impedances between the electrode array and tissue. In this way, the charge imbalance issue that might be attributed to unipolar monophasic voltage inducing could be avoided. The voltage regulator in the stimulation circuit was able to generate a constant 20 V with a minimum pulse width of 1 μs. The stimulation frequency can be as high as 250 stimulation pulses per second when periodic stimulation pulses are employed. The microcontroller can precisely control the voltage to a current converter circuit to issue pulses with the parameters above. The biphasic stimulation current is at the level of 40 μA with a 5-MΩ impedance electrode. Stimulation with different parameters and purposes can be issued while brain activities are being recorded at the same time. The high voltage converter will be shut down when no stimulation is to be delivered in order to reduce power consumption. Based on the brain activity report, the system can be used by researchers to induce stimulation and monitor brain response to stimulation. The stimulation module we developed may not outperform some state of the art stimulators; however, as an integrated system with a compact size and multi-functionalities, the stimulation module is adequate as part of the BBMI system.

## 4. Wireless Power Transfer and Power Budget

Many conventional implantable devices use a primary battery with a fixed lifespan for their power source. Wireless power transmission is an attractive alternative to batteries in low-power biomedical implants and has received increasing research interest in recent years [33,34]. Radio frequency (RF), ultrasound [15,35], infrared light and low-frequency magnetic field are considered as viable wireless power transfer options. Wireless power charging technologies, such as magnetic resonance and induction coupling, have limited applications because of their short transfer distance compared to device size and the magnetic field intensity limitation for the safety of body exposure. As an alternative, the biocompatible wireless power transfer using ultrasonic resonance developed in a collaborating study offers a smaller circuit size and no heat dissipation. In this study, we used the wireless power transfer technique fully described in [15] to deliver power to the implantable BBMI system as demonstrated in Figure 3. Also illustrated in Figure 3, ultrasonic wireless power transmission is composed of converting electrical energy to ultrasonic energy and vice versa. To achieve an efficient power transfer ratio, we calculate the optimal transfer frequency of the ultrasound based on the acoustic radiation and damping effect. The optimal load resistance is also determined to match with the power condition of the ultrasound receiver. The transfer frequency of the transmitter is determined to match the calculated optimal transfer frequency. Since the wireless BBMI system we proposed would be placed as a skull implant, the scalp is the only object that lies between the ultrasonic transmitter and the receiver. The detailed implementation of the ultrasonic power transmitter and the receiver is presented in our previous work [15]. The key parameters of the novel ultrasonic power transfer module are listed in Table 1. In this study, we provide the energy harvesting module that integrates the receiver of the ultrasonic power transfer unit with the power management module of the BBMI and test the system performance.

The sensor module was designed to operate using a coin-type rechargeable battery with a 30-mm diameter. To extend the lifetime of the system between charges, an elegant power management scheme was employed to turn off components when they are not active to conserve battery. Transmit power is one of the radio characteristics that affects power consumption in wireless communication systems. The *μ*ESB protocol supports radio solutions with transmit power ranging from −20 dBm–4 dBm. A separate experiment was conducted to evaluate different transmit powers, and a 6.22% increase in power consumption was observed when transmit power changed from −20 dBm–0 dBm. Thus, −20 dBm was applied for bench test, while 4 dBm was used for in vivo testing. The sampling rate is another factor that influences the power consumption; the total power consumption of the data acquisition module depends on different configurations. There is an increase of 212% in power consumption when we increase the sampling rate from 1 kSPS–30 kSPS. Since the primary interest in the first stage of the experiment is planned to record and analyze LFPs, we choose 1 kSPS as our sampling rate and monitor the brain waves in the low frequency band.

## 5. Experiment Setup and Results

To validate the recording and stimulation circuit, as well as the system performance, both bench tests and in vivo tests were performed as illustrated in the following subsections.

### 5.1. Bench Test

The system is able to transmit data at three different transmission rates. For the bench test, we set the on air data rate to be 1 Mbps. On the receiver side, the baud rate was set to 1 Mbps. With 16 channels enabled, we were able to achieve an 800 SPS data rate with the ESB protocol. A user interface was designed with Python to read and plot data through a serial port.

**Figure 4:**
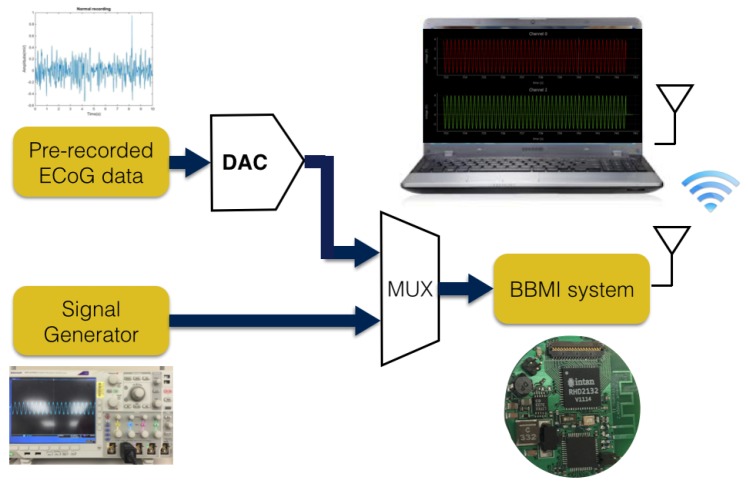
Experimental setup for the bench test with two input sources.

Two different input sources were fed into the device for the bench test as shown in Figure 4. The input signal range for the front-end amplifiers of our device is ±5 mV. One test source was a sine wave with an amplitude of 3.3 mV; the frequency changed dynamically from 1 Hz–100 Hz. Another test source was a set of pre-recorded intracortical signals recorded from an animal model using a research-grade recording system. The intracortical data stream was first fed into a digital-analog converter (DAC) and then to the input of the BBMI device. All data received on the host side were plotted in real time and saved to a .csv file locally for validation. The stimulation module was validated by using a scope to monitor the output stimulus amplitude and frequency.

### 5.2. Power Analysis

**Figure 5:**
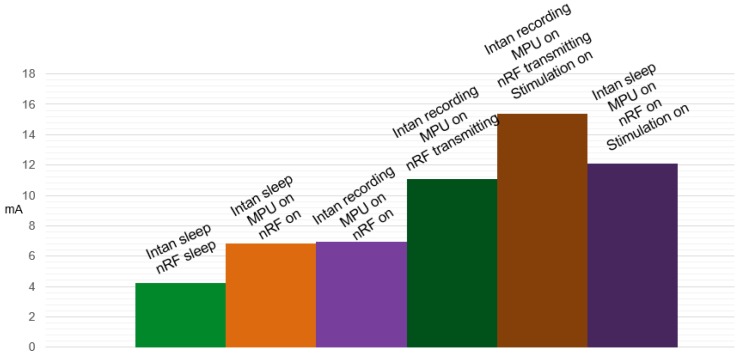
Current consumption under different system configurations.

To examine the power consumption with a given configuration in a laboratory environment, accurately and precisely, we monitored the energy consumed by the device with different combinations of submodules enabled, as shown in Figure 5. When the sensor first turned on, recording, MPU and the wireless link are in sleep mode, which consumes a baseline current of 4.244 mA. Once we activate the MPU and wireless link module, which is the nRF51822 chip, the current consumption of the whole system becomes 6.851 mA. Further, if the data recording module is enabled for 16 channels with fully-functioning amplifiers and ADC, a slight increase can be observed with a total current consumption of 6.940 mA, since the RHD2132 chipset is an extra-low power solution targeted for implantable solutions. By enabling the transmission protocol for continuous data transmission at 800 SPS, the current consumption becomes 11.10 mA. For BBMI with the stimulation module enabled and the stimulation issued every 1.25 ms with a pulse width of 100 µs, the measured current consumption is 15.40 mA. This is the total current consumption with all of the function blocks enabled and performing with full efficiency for the BBMI system we presented. Finally, Table 2 gives the breakdown of the current consumption for each component in the BBMI system.

The sensor is powered by one Li-ion rechargeable battery, which has a nominal capacity of 200 mAh and a nominal voltage of 3.7 V. As a result, it can provide about 10 h of working time without recharging the battery.

When the battery drained out, the ultrasonic power transfer system is activated to recharge the battery. An experiment to measure the charging time and efficiency of the system was conducted by placing the transmitter (TX) on top of receiver (RX). In between, a 2-mm layer of ultrasonic cavitation gel was applied to simulate human skin. By adjusting the frequency and amplitude accordingly, we observed the best efficiency to be about 20% with a 470-kHz frequency and a 20-Amps amplitude. To charge a fully-drained rechargeable battery, the charging voltage is about 4.2 V, and the charging current is around 26 mA. It takes the whole system approximately 4.86 h to be fully charged.

### 5.3. In Vivo Test

Brain recordings can be broadly classified into two categories; the signals below 300 Hz are called LFPs and relate to increased brain activity in a particular area, while signals above 1000 Hz provide more detailed information on neural spikes [36]. To record neural spikes, the sampling rate should be set to 15 kSPS and above, while for LFPs acquisition, lower sampling rates are adequate. Using intracortical electrode arrays, single unit activity (SUA), multi-units activity (MUA) and LFP can be recorded while cortical surface electrodes enable recording of LFP and ECoG from the surface of the brain. LFP records the field electrical potential from a small group of neurons. It records sensorimotor rhythms similar to ECoG, but with higher spatial resolution. Further, in order to analyze the integrative synaptic processes, LFP is the signal of interest instead of spikes, because synaptic processes cannot be captured by spike activity of a small number of neurons. During the past few decades, LFP has been used to study higher level cognitive processes involving attention, memory and perception [37,38,39], as well as to control prosthetic devices [40,41,42]. The LFP is also a promising indicator for monitoring neural activity, since the signal can be captured more easily and is more stable in chronic settings when compared to spikes [43].

Thus, in this study, in vivo tests of the system were performed on a behaving monkey to record his LFP oscillations. The system was tested by using electrodes implanted in the left hemisphere primary motor cortex area to observe LFP oscillation that related to hand motion. The Utah Array was connected to the external recording and stimulation units via a connector installed on the skull during these measurements. The front-end amplifier lower bandwidth was set to 0.1 Hz, and the higher bandwidth was set to 1 kHz. An on board high-pass filter was enabled with a cutoff frequency of 0.3 Hz to remove the residual DC offset voltages.

**Figure 6:**
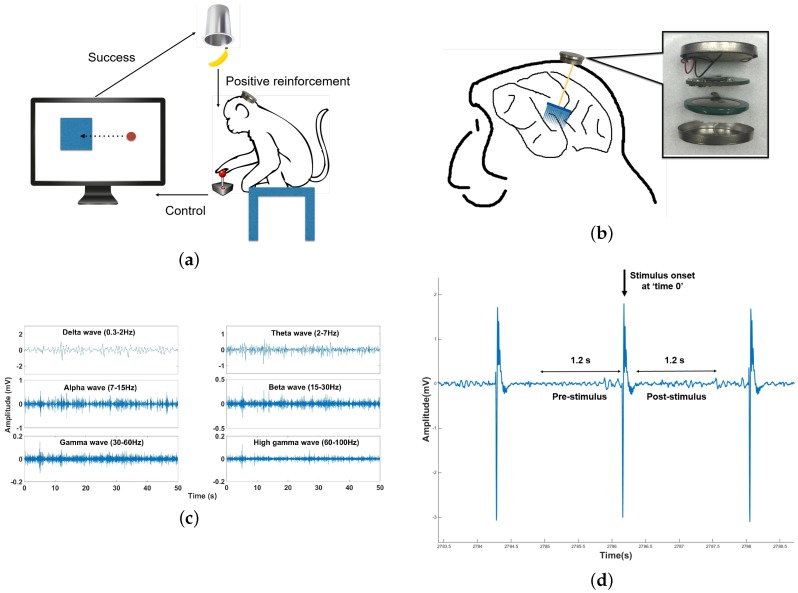
(**a**) Experimental setup for the in vivo test. (**b**) Implantable BBMI module connection to the Utah Array. (**c**) Different brain rhythms of the local field potential (LFP) recorded from the Monkey’s motor cortex. (**d**) Recorded stimulation artifacts.

The experimental setup is illustrated in Figure 6a,b. The monkey was securely sitting in front of a PC monitor playing a game. A specific visual cue was presented on the monitor; in this case, a rectangular box would pop up randomly on the screen. The monkey was instructed to move the cursor on the screen towards the rectangular box by controlling a joystick. If the monkey successfully moved the cursor into the rectangular area, he was rewarded with treats. During the experiment, all data were first applied with a fourth-order low-pass Butterworth filter with a cutoff frequency of 100 Hz. Then, for the normal LFP recorded for 1000 s in the first session, signals were band-passed into different rhythms, as shown in Figure 6c. Six casual fourth-order Butterworth band-pass filters were applied to the data stream with cutoff frequencies of 0.3 Hz–2 Hz (delta wave), 2 Hz–7 Hz (theta wave), 7 Hz–15 Hz (alpha wave), 15 Hz–30 Hz (beta wave), 30 Hz–60 Hz (gamma wave) and 60 Hz–100 Hz (high gamma wave).

**Figure 7:**
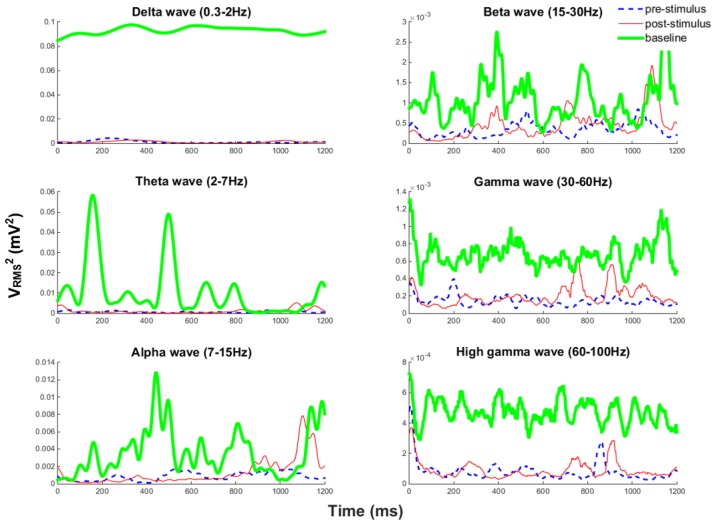
Averaged pre-stimulus and post-stimulus band power changes compared with the no stimuli baseline power.

In the next part of the experiment, the stimulation module was tested by issuing stimulation pulses with a biphasic current rating of 40 μA; stimulus was issued every 1.6 s. Stimulation artifacts could be observed from the recording data, as shown in Figure 6d. The 1.2-s data segments were chosen outside the time window of the artifact, because if we tried to remove the artifacts by applying additional filters, it is possible that the some parts of the information from the original data may be removed, as well. Thus, we chose the safest and easiest way by analyzing the data outside the window of the artifact. Based on the illustration in Figure 6d, Figure 7 shows the stimulus-triggered averaged signal band power changes 1.2 s ahead of when the stimulus was issued and 1.2 s after the stimulus was issued. The stimulus was issued at Time 0, and the pre-stimulus has been plotted in reverse time. At the point when the stimulus was issued, the brain signal took about 200 ms to recover. However, during the analysis, we took the whole 200-ms recovery period as “Time 0”; thus, the pre-stimulus is from t=−1.2–0, and the post-stimulus is from t=0–1.2 s.

To estimate the power for different frequency bands, first, we pick one stimulus and analyze the pre-stimulus and post-stimulus data segments. A sliding window has been applied to each frequency band in each data segment, and the window size is 20 samples. Inside the window, $V_{RMS}^{2}$ has been calculated as a representation of power over time. The average power change is obtained by randomly picking 10 stimuli from the beginning of the stimulation session, in the middle of the stimulation session and at the end of the stimulation session, and then calculating the mean $V_{RMS}^{2}$ obtained from the 10 stimuli data segments. Both pre-stimulus and post-stimulus were compared with the baseline band power when no stimulation was presented. Baseline power is calculated by dividing the 1000 s of recorded data before stimulation session into 1.2-s segments and obtaining the average power over those segments. The blue dashed line represents pre-stimulus; the red thin solid line shows post-stimulus; and the green thick line is the baseline power with no stimuli involved. We could see, by introducing periodic stimuli, an energy decrease in Delta , Gamma and high Gamma bands were observed; but no significant energy changes for Theta , Alpha and Beta bands.

**Figure 8:**
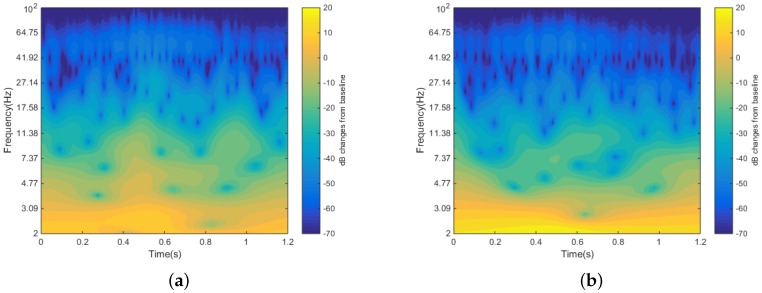
(**a**) First stimulus dB change from the baseline. (**b**) Last stimulus dB change from the baseline.

Figure 8 shows the time-frequency power changes from the first stimulus pulse to the last stimulus pulse. We use the decibel ratio to represent the strength of the target signal in comparison with the baseline level of power in the same time and frequency domain.

(1) $$dB_{tf}=10\times \log10\left(\frac{activity_{tf}}{\bar{baseline_{f}}}\right)$$

where the overhead bar in $\bar{baseline_{f}}$ indicates the mean across the baseline time period, and *t* and *f* are the time and frequency points. In Figure 8a, the activity time period is the signal recorded 1.2 s after the first stimulus, and the baseline is the 1.2-s data points before the first stimulus is issued. In Figure 8b, the activity time period is the signal captured 1.2 s after the last stimulus, and the baseline is the same as Figure 8a. There is a significant power increase right after the stimulus onset in the Delta band and Theta band, while in the Alpha band and Beta band, the power decreases from the first stimulus to the last stimulus about 400 ms after the stimulus onset.

**Figure 9:**
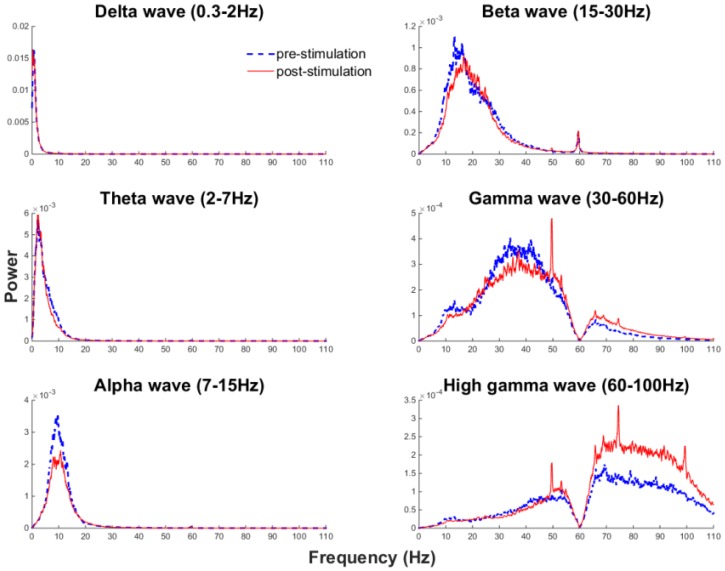
Pre-stimulation and post-stimulation power spectrum changes for each frequency band.

After the stimuli were issued periodically for 1000 s, normal LFP without stimulation was recorded again for another 1000 s. The LFPs from pre-stimulation and post-stimulation sessions were compared in the frequency domain, as shown in Figure 9. By calculating different frequency band powers, an increase in the power spectrum after the stimulation session in high frequency oscillations (beta and high gamma) had been observed. Specifically, around 20 Hz was the cutoff frequency point where energy below this cutoff frequency did not increase, but energy higher than the cutoff frequency had apparently increased in the beta band. Previous research has shown the Beta oscillations in the primary motor cortex to be involved in steady arm and hand motions [44,45,46,47,48]; the stimuli applied during our experiment act as a reinforcement for arm and hand motion as evidenced by an increase in the energy spectrum in high oscillation bands associated with sensorimotor motion. The changes in the specific LFP spectrum might be subject to the corresponding changes in the behavioral state, such as arousal, triggered by the stimuli. Though the exact interpretation of the observed oscillation changes can be further discussed, either case indicates that the stimulation circuit is working as expected.

Table 3 provides a comparison of the system presented in this paper with state of the art technology systems published recently; we have limited the comparison to studies that implemented BMI using COTS components and published in recent years only. Compared to most recent BBMI systems that emerged in 2015, the system presented in this paper has several advantages as reported in Table 3. It offers the smallest form factor, while providing similar or better functional capabilities than offered by other works in one single 30-mm PCB design. Compared with works presented in [7,11], which have similar function blocks implemented, but in separate PCB boards with a larger total size, the system we presented integrates functions including neural signal recording, neural stimulator, wireless data and power transfer into one compact system that avoids excessive surgeries for implantable devices. The number of channels for recording and stimulation is adequate for most clinical applications and exceeds most of those reported in the literature. The power consumption is comparatively low for brain implants developed using COTS hardware components with respect to other works listed in the table. A novel wireless power transfer technique is integrated into the system. Communication between devices and the host is completely wireless by using a proprietary protocol offering up to 2 Mbits/s.

## 6. Discussion and Conclusion

In this study, we developed a small form factor wireless BBMI system for cortical and spinal plasticity. The system recorded signals from neural tissue via implanted MEAs, provided on board processing for real-time decoding of the signals and executed a prescribed stimulation pattern. The system communicates with a host system using a high speed, low power wireless communication channel. The system is powered via a rechargeable battery that can be charged wirelessly using an ultrasonic power transfer module. With a fully-charged battery, this small form factor system can operate up to 10 h, which is sufficient for a long-term experiment in the laboratory environment. The implanted system has very tight requirements on power consumption, as well as the data rate. However, one must seek a balance between those two major factors, since a higher data rate usually leads to higher power consumption. In order to solve this conflict, this system also provides an on board signal processing capability to reduce the data rate without sacrificing the amount of information transferred over the communication channel. The stimulation module contributes greatly in terms of power consumption. In [49], a fully-implantable stimulator with two channels of output providing a constant mono-phase voltage pulse (50 mV–3 V) with a 0–200-Hz pulse frequency and a 400–1200-μs pulse width has been presented. The stimulation module integrated in our system needs to find a balance between power consumption and full function. Thus, we exclude the selectable constant voltage function for better device size and lower power consumption. However, the stimulation module we designed is suitable for high voltage stimulation with a 20-V voltage output, which ensures enough stimulation current for brain and spinal stimulation. In the next version of our BBMI system, we will include bi-phase current stimulation with selectable current values. The wireless BBMI system developed in this study provides a chronic and reliable solution for a long-term laboratory animal experiment using 32 channels of recording and four channels of stimulation. The system is aimed at understanding cortical plasticity and creating new pathways in the brain. Understanding how the brain functions and creating bridges between neurons may boost solutions for people with function loss or severe CNS disease. The primary goal of this stage of in vivo testing is to test the circuitry functionality. Work is in progress to package the system in a biocompatible titanium case to be part of the skull and serve as a fully-implanted system with both recording and stimulation functions. Meanwhile, we will continue to work on the protocols to perform online spike detection and to demonstrate the changes in different spectral bands related to monkeys’ behavior.

## Figures and Tables

**Table 1:** Key parameters for ultrasonic wireless power transfer across 10-mm pig skin [15].

Parameters	Value
**Driving frequency**	1.056 MHz
**Maximum power transfer**	250 mW
**Maximum efficiency**	22.5%
**Charging time (200 mAh battery)**	4.88 h
**Temperature change**	+1.4 ℃

**Table 2:** Breakdown of the current consumption for the implantable BBMI module.

Components	Current consumption (mA)
**Baseline**	4.244
**MPU**	2.607
**Wireless link**	4.100
**Recording**	0.089
**Stimulation**	4.300
**Total**	15.34

**Table 3:** Comparison of state of the art works. SPS, samples per second; ESB, Enhanced ShockBurst.

	This Work	Neurochip-2	WIMAGINE	PennBMBI	W-HERBS	Nguyen	Angotzi
	(2015)	(2011) [9]	(2015) [8]	(2015) [11]	(2011) [7]	(2014) [10]	(2014) [12]
**Recording channel**	32 unipolar/bipolar	3 unipolar/bipolar	64	4	64×2	32	8
**Gain**	192	1000 or 5000	1000	200	40–80 dB	200	-
**ADC resolution**	16 bits	8 bits	12 bits	12bits	12 bits	16 bits	8 bits
**Sampling rate**	800 SPS/channel up to 30 kSPS/channel	256 SPS	1 kSPS	21 kSPS	1 kSPS	400 kSPS 12.5 kSPS/channel	15 kSPS/channel
**Bandwidth**	0.1–20 kHz	10 Hz–7.5 Hz	0.5 Hz–400 Hz	0.05 Hz–6 kHz	0.1 Hz–1 kHz	0.2 Hz–5 kHz	1 Hz–10 kHz
**Communication**	2.4 GHz RF communication link with *μ*ESB protocol	Serial cable/ infrared data link	Proprietary UHF link in MICS band	2.4 GHz RF communication link	Bluetooth	Wired	2.4 GHz ISM band
**Power supply**	Rechargeable 3.7 V battery	1 or 2 rechargeable 3.6 V battery	Inductive link	Rechargeable 3.7 V battery	Polymer Lithium ion 3.7 V battery	5 V USB	3.7 V 700 mAh battery
**Power consumption**	4.22–15.4 mA	284–420 mW	75 mW w/o charging 350 mW w/ charging	7.3 mA transmit for sensor node only	4.9 mW (AFE) 300 mW (wireless)	-	-
**Battery charging**	Wireless ultrasonic charging	-	-	-	Wireless charging coil 4 W at distance 38 mm	-	-
**Size**	35 mm in diameter 10 mm in thickness	63×63×30 mm	50 mm in diameter 12.54 mm in thickness antenna:10 cm${}^{2}$	56×36×13 mm + 43×27×8 mm + 31×13×8 mm	20×30×2.5 mm + 60×60×8 mm + 40 mm in diameter 8 mm in thickness	29.5×43.4 mm	-
**Stimulation channel**	4 unipolar/bipolar	3 unipolar/bipolar	-	2	-	1 optical stimuli	8 bipolar
**Stimulation voltage**	20 V	±15 V (normal) ±50 V (high V)	-	±12 V	-	-	±9 V
**current intensity**	40 μA	10–200 μA current pulse or 0.5–5 mA	-	0–1 mA	-	-	300 μA
**Pulse width**	1 us minimum	0.2 ms, 0.6 ms	-	200 μs	-	-	-
**Pulse frequency**	250 Hz	1/min or 1/10 min	-	-	-	-	-
